# Supplementary material for: Enhanced ResNet-50 for garbage classification: Feature fusion and depth-separable convolutions
Source: PLoS One. 2025 Jan 27;20(1):e0317999. doi: 10.1371/journal.pone.0317999 (PMC11771864; doi:10.1371/journal.pone.0317999)
Supplement: Attachment — Submitted filename: PONE-D-24-42358_reviewer.docx [file pone.0317999.s001.docx]

**Improving the Performance of ResNet-50 Model for Garbage Image Classification: Redundancy-Weighted Feature Fusion and Depth-Separable Convolution Strategies**

#Abstract: The abstract is well composed stating the project problem, a approach to the problem identified, and solution proffered.

\#	Introduction: There is a need for clarity in the exiting model and the proposed model. In the last paragraph of the introduction section. “The paper proposed ResNet-50 Model.”

In Table 2. “ResNet-50 [33]” indicating the model was used by this author(s). Also, on the same Table there is a row with “Ours”. The question is how do you arrived to “Ours” model? Much should be done in the area of parameters for better understanding to researchers in other field of study.

Also, the issue needs to be redressed for better understanding of the authors contributions to the body of knowledge.

#Figures: There is a need for the authors to improve the figures attached resolutions for clarity.
