# Supplementary material for: Enhanced ResNet-50 for garbage classification: Feature fusion and depth-separable convolutions
Source: PLoS One. 2025 Jan 27;20(1):e0317999. doi: 10.1371/journal.pone.0317999 (PMC11771864; doi:10.1371/journal.pone.0317999)
Supplement: Attachment — Submitted filename: Response to Reviewers.docx [file pone.0317999.s002.docx]

**Original Manuscript ID:** PONE-D-24-42358

**Original Article Title:** “Improving the Performance of ResNet-50 Model for Garbage Image Classification: Redundancy-Weighted Feature Fusion and Depth-Separable Convolution Strategies”

Dear Editor,

Thank you for allowing a resubmission of our manuscript, with an opportunity to address the reviewer's comments.

Best regards,

<Yuheng Ren> et al.

**Academic editor, Concern:** Specifically, please make sure the literature review includes most recent relevent works. You may cite the works recommended by the reviewers only if you feel these align well with your work. Moreover, the findings of the study and the contributions may be elaborated further to improve the tutorial content of the manuscript.

**Author response:**

Dear Academic Editor,

Thank you for your careful review and valuable suggestions.

We have updated the literature review section to include the most recent and relevant research, ensuring our work aligns with the latest developments. Regarding your suggestion to elaborate further on the findings and contributions of the study, we have added relevant content in the revised version, providing a more detailed explanation of our results and innovations to enhance the manuscript's tutorial value and readability.

We believe these revisions will strengthen the academic quality and practicality of the manuscript. Thank you for your thoughtful guidance. We will continue to strive to meet the high standards of the journal.

Once again, we sincerely appreciate your time and effort, as well as your support and valuable feedback on our research. We look forward to your final review and hope for your approval.

**Reviewer#1, Concern#1:** In the literature review, the authors are advised to add some updated methods from 2022-2024 to provide an up to date overview.

**Author response:** Thank you for your valuable suggestions. We highly appreciate your advice and have made the corresponding updates and additions to the literature review section. During the 2022-2024 period, we found that there are relatively few studies on deep learning-based garbage image classification models using the TrashNet dataset. Therefore, we have added two representative papers, which are:

*[50] Shi C, Tan C, Wang T, et al. A waste classification method based on a multilayer hybrid convolution neural network[J]. Applied Sciences, 2021, 11(18): 8572.*

*[51] Hossen M M, Majid M E, Kashem S B A, et al. A reliable and robust deep learning model for effective recyclable waste classification[J]. IEEE Access, 2024.*

At the same time, we noticed that there has been more progress in deep learning-based garbage image classification models using self-built datasets. Therefore, we have included the following papers to enrich the review:

*[52] Alsubaei F S, Al-Wesabi F N, Hilal A M (2022). Deep learning-based small object detection and classification model for garbage waste management in smart cities and IoT environment. Applied Sciences, 12(5): 2281.*

*[53] Liu W, Ouyang H, Liu Q, et al. (2022). Image recognition for garbage classification based on transfer learning and model fusion. Mathematical Problems in Engineering, 2022(1): 4793555.*

*[54] Li Y, Liu W (2023). Deep learning-based garbage image recognition algorithm. Applied Nanoscience, 13(2): 1415-1424.*

We believe these newly added references provide readers with a more comprehensive and up-to-date overview of the research trends. If you think there are other important studies or areas that should be included, we are happy to continue making improvements to ensure the completeness and timeliness of the literature review.

**Reviewer#1, Concern#2:** In deep learning section, the authors are advised to add necessary introduction related to deep learning and its importance by citing the recent deep learning models such as iAFPs-Mv-BiTCN, AIPs-DeepEnC-GA, DeepAVP-TPPred, PAtbP-EnC, Deepstacked-AVPs, and AIPs-SnTCN for the readers concern to provide broader overview.

**Author response:** Thank you for your valuable suggestions on the deep learning section of our manuscript! In the paper, We added background on deep learning under the sub-chapter “Garbage image classification method based on convolutional neural network” and further elaborated on the importance of deep learning. At the same time, we have also cited some recent deep learning models, such as iAFPs-Mv-BiTCN, AIPs-DeepEnC-GA, DeepAVP-TPPred, PAtbP-EnC, Deepstacked-AVPs, and AIPs-SnTCN, etc., to provide readers with a more comprehensive perspective. Finally, it naturally leads to convolutional neural networks and the research on garbage image classification based on convolutional neural networks in recent years.

We believe these additions will improve the clarity and depth of the deep learning section, provide a solid background for readers less familiar with the field, and highlight the cutting-edge research on which our work is based.

**Reviewer#1, Concern#3:** In order to provide deep understanding of the model, the authors are suggested to provide SHAP analysis based feature analysis to highlight the high contributory features that might be helpful for future real life implementation.

**Author response:** We sincerely appreciate your valuable suggestions and the detailed review of our work. We fully agree with your perspective on providing a deeper understanding of the model, as this is crucial for enhancing its interpretability and reliability in real-world applications.

Regarding your suggestion to use SHAP analysis to highlight the high-contributory features in the model, we would like to explain our reasoning. We considered several feature importance analysis methods and ultimately chose Grad-CAM because it is a widely recognized technique in image classification tasks that can intuitively highlight the regions of the image that the model focuses on during the classification process. The basic principle of Grad-CAM is to compute the contribution of each feature map to the final classification result using the gradient information from the neural network. Specifically:

1. Compute the gradient: For the input image X, we calculate the gradient of the last convolutional layer through backpropagation.
2. Weight the feature maps: We multiply each feature map's gradient by its corresponding activation value, obtaining each feature map's contribution to the final classification.
3. Generate the Grad-CAM heatmap: We multiply the weighted feature maps by their activation values and sum them up to generate a heatmap. Then, we apply a ReLU activation to the heatmap to retain only the positive contribution and remove negative areas.
4. Overlay the heatmap on the original image: The generated heatmap is resized to match the size of the original image and overlaid on top of it. This allows us to visually see which areas of the image have the most influence on the model’s decision.

In the heatmap, the red or yellow regions represent the areas the model focuses on, which significantly impact the model’s classification prediction. Since the principles of Grad-CAM are widely accepted and easy to understand, we did not discuss the details of its theory in the paper.

We have added an experimental section. Specifically, we randomly selected 12 images from the TrashNet test set, generated the corresponding heatmaps using Grad-CAM, and overlaid them on the original images to visually demonstrate the key areas the model focuses on during the classification process, as shown in Fig.5. It can be seen that the proposed model effectively focuses on the objects in the image, demonstrating its ability to capture critical information, thereby achieving high classification accuracy.

**Fig.5 The heatmap of the model's focused regions based on Grad-CAM.**

**Reviewer#1, Concern#4:** How the authors evaluate the overfitting and generalization of the proposed model.

**Author response:** Thank you for your attention to our model's overfitting and generalization ability. In response to your concerns, we provide a detailed explanation of our evaluation methods and results.

Overfitting typically manifests as the model performing very well on the training set (i.e., with low loss and high accuracy) but performing poorly on the test set (i.e., with high loss and low accuracy). However, as shown in Fig. 6, our model maintains an accuracy of around 94% on the test set during the later stages of training, indicating that it performs well in classifying the test set. The loss stabilizes around 0.25, which is relatively low for a classification task and suggests a low error rate. The stability of the loss aligns with the model's performance on the test set, indicating that the proposed model does not exhibit significant signs of overfitting.

**Fig.6 (a)Loss curves of different models on TrashNet dataset. (b)Accuracy curves of different models on TrashNet dataset..**

As described in the paper, we conducted an additional experiment to evaluate the model's generalization ability further. We occluded different regions of an original image, creating nine distinct sub-images. The accuracy of our model on these occluded sub-images is consistently higher than that of ResNet-50 and ResNet-50-B, with accuracy remaining around 90%, as shown in Fig. 9. This result demonstrates that our model can maintain high recognition accuracy even when critical region information is missing, highlighting its strong generalization capability.

**Fig.9 The accuracy of three models on the nine new test sets.**

We believe these evaluation results adequately demonstrate our model's generalization ability and resistance to overfitting. We appreciate your suggestions and look forward to your feedback on the above explanation.

**Reviewer#1, Concern#5:** An up to date comparison of the proposed model with existing state of the art models will be necessary.

**Author response:** Thank you for your valuable suggestion. Regarding your recommendation to compare the proposed model with existing state-of-the-art models, our response is as follows:

As mentioned in our response to your second question, we have reviewed the relevant literature and found that there has been relatively little deep learning-based research on garbage image classification using the TrashNet dataset in recent years. Therefore, the results of the two documents mentioned in the answer to the second question on the TrashNet data set and the document [49] previously cited in our paper have been included in Table 4 for comparison.

We fully agree that comparing our work with the latest models is crucial for evaluating its novelty and performance. To this end, we have incorporated the most recent research as comprehensively as possible to ensure that our model is fairly evaluated within the current research context. We believe the comparison in Table 4 covers the major studies related to the TrashNet dataset that are currently available.

We look forward to your further guidance and valuable suggestions.

**Reviewer#1, Concern#6:** What should be the future directions of the proposed model.

**Author response:** Thank you for your suggestion regarding the future direction of the proposed model. In response to your feedback, we have added a section in the “conclusion” that briefly outlines potential future work. We look forward to your further feedback and greatly appreciate your valuable input.

Again, we would like to express our sincere gratitude for your time and effort, as well as your support and valuable feedback on our research. We look forward to your final review and hope to receive your approval.

**Reviewer#2, Concern#1:** Abstract: The abstract is well composed stating the project problem, a approach to the problem identified, and solution proffered.

**Author response:** Thank you for your positive feedback on the abstract. We are glad that it effectively conveys the project problem, the approach identified to address it, and the proposed solution. We appreciate your recognition of the clarity and structure of the abstract.

**Reviewer#2, Concern#2:** Introduction: There is a need for clarity in the exiting model and the proposed model. In the last paragraph of the introduction section. “The paper proposed ResNet-50 Model.”

**Author response:** We appreciate your insightful comments. In response to your suggestion, we have revised the last paragraph of the introduction and also made corresponding modifications to the abstract to highlight the distinction between the existing models and the one proposed in this paper. To avoid overlap with the contributions and changes made in the abstract, the revision of the last paragraph of the introduction is a general summary. The specific changes are as follows:

*This paper proposes a new garbage image classification model that uses ResNet-50 as the backbone network. In this model, a redundancy-weighted feature fusion module is proposed, combined with depth-separable convolution techniques to optimize ResNet-50. These two techniques effectively reduce the number of model parameters while improving classification efficiency. Additionally, the standard Focal Loss is weighted to mitigate the impact of class imbalance on model performance, enhancing the model's robustness. Experimental results on the TrashNet dataset show that the proposed model significantly improves classification accuracy and robustness while maintaining fewer parameters and faster detection speed.*

We hope this revision addresses your concerns and more clearly conveys the distinction between our proposed and existing models.

**Reviewer#2, Concern#3:** In Table 2. “ResNet-50 [33]” indicating the model was used by this author(s). Also, on the same Table there is a row with “Ours”. The question is how do you arrived to “Ours” model? Much should be done in the area of parameters for better understanding to researchers in other field of study. Also, the issue needs to be redressed for better understanding of the authors contributions to the body of knowledge.

**Author response:** We greatly appreciate your thorough review and valuable feedback on our paper. In response to your suggestions, we have made the following revisions:

1. In the “abstract”, we have provided a clearer description of the proposed model and explicitly pointed out this paper's innovations to help readers better understand our approach.
2. In the final paragraph of the “introduction”, we have provided a general summary, further strengthening the description of the proposed model and emphasizing the innovations once again.
3. In the second paragraph of the “Proposed Method” section, we have added detailed explanations of the model structure and parameters and the corresponding table to help readers better understand our model.

We believe these revisions address your suggestions and improve the completeness and readability of the paper. Below is the newly added section on the model structure and the parameter table for your reference:

**Reviewer#2, Concern#4:** Figures: There is a need for the authors to improve the figures attached resolutions for clarity.

**Author response:** Thank you for your attention and suggestions regarding the clarity of the figures. We fully agree with your request and have adjusted and optimized the figures' resolution to ensure readers can better understand the information presented in the images. We believe these improvements will further enhance the quality of the paper.

Again, we would like to express our sincere gratitude for your time and effort, as well as your support and valuable feedback on our research. We look forward to your final review and hope to receive your approval.

**Reviewer#3, Concern#1:** The title is clear and relevant but could be more concise by removing redundant phrases like “Improving the Performance of.” For example, “Enhanced ResNet-50 for Garbage Classification: Feature Fusion and Depth-Separable Convolutions” might be more impactful.

**Author response:** Thank you for your valuable suggestion. We appreciate your emphasis on the title's conciseness and agree with your point. Following your recommendation, we have revised the title to “Enhanced ResNet-50 for Garbage Classification: Feature Fusion and Depth-Separable Convolutions” to improve its conciseness and impact.

**Reviewer#3, Concern#2:** The abstract effectively summarizes the study's objectives and findings but lacks specific quantitative results (e.g., accuracy improvement percentages). Including these would strengthen its impact and clarity.

**Author response:** Thank you for your valuable feedback. In response to your suggestion, we have revised the abstract to include specific quantitative results. Specifically, we have updated the abstract to state:

*Experimental results on the TrashNet dataset show that the proposed model effectively reduces the number of parameters, improves detection speed, and achieves an accuracy of 94.14%, surpassing the vast majority of existing deep learning-based waste image classification models, demonstrating its solid practical value*.

We believe this revision enhances the clarity and impact of the abstract.

**Reviewer#3, Concern#3:** The introduction provides a strong rationale for the study by highlighting the environmental importance of waste classification and the limitations of existing methods. However, it could better articulate the novelty of this approach compared to prior works.

**Author response:** Thank you for your constructive feedback. Based on your suggestion, we have revised the final paragraph of the introduction and made corresponding changes to the abstract to highlight the novelty of the proposed model better. To avoid redundancy with the contributions and revisions in the abstract, the changes to the final paragraph of the introduction provide a concise summary, as detailed below:

*This paper proposes a new garbage image classification model that uses ResNet-50 as the backbone network. In this model, a redundancy-weighted feature fusion module is proposed, combined with depth-separable convolution techniques to optimize ResNet-50. These two techniques effectively reduce the number of model parameters while improving classification efficiency. Additionally, the standard Focal Loss is weighted to mitigate the impact of class imbalance on model performance, enhancing the model's robustness. Experimental results on the TrashNet dataset show that the proposed model significantly improves classification accuracy and robustness while maintaining fewer parameters and faster detection speed.*

We hope this revision addresses your concerns and better conveys the novel aspects of our work.

**Reviewer#3, Concern#4:** The literature review is comprehensive and well-structured, offering a broad overview of related works in machine learning and CNN-based garbage classification. However, it lacks critical analysis of gaps in prior studies that this research addresses.

**Author response:** We sincerely appreciate your recognition of our literature review and your valuable suggestions. We highly value your concern regarding the lack of critical analysis of the gaps in prior research. In response, we have added two paragraphs at the end of the subsections "Garbage Image Classification Methods Based on Machine Learning" and "Garbage Image Classification Methods Based on Convolutional Neural Networks." In these paragraphs, we discuss existing methods' challenges in classification accuracy, model parameters, detection speed, model generalization ability, and adaptability to real-world application scenarios. Furthermore, we have revised the last paragraph of the introduction and the abstract, where we explicitly highlight how the method proposed in this study addresses these gaps with improvements and innovations. We hope these revisions will enhance the paper's overall quality.

**Reviewer#3, Concern#5:** The methodology is detailed and includes innovative contributions such as redundancy-weighted feature fusion and depth-separable convolutions. However, some technical aspects (e.g., how weight coefficients are learned) require further clarification for reproducibility.

**Author response:** We sincerely appreciate your detailed review of our methodology and your recognition of our work's innovative contributions. In response to your suggestion for further clarification of the technical details, we have focused specifically on the description of the weight coefficient learning process. In the revised manuscript, we have added further explanations in the relevant sections to ensure that these key steps are presented more clearly and comprehensively, thereby enhancing the reproducibility of the research. We look forward to your continued guidance and feedback.

**Reviewer#3, Concern#6:** Results are presented with sufficient detail but could benefit from additional visualizations (e.g., confusion matrices or feature maps) to illustrate model performance across categories. Comparative analysis with state-of-the-art methods should be expanded.

**Author response:** We sincerely thank you for your valuable feedback. Based on your suggestions, we have made the following improvements in the revised version:

First, as described in the paper, we compared the classification accuracy of ResNet-50, ResNet-50-B (the best-performing model based on ResNet-50), and our proposed model across six categories of garbage in the TrashNet dataset. The results are shown in Fig. 7. As seen in Fig. 7, our model achieves significant improvements in classification accuracy across all six categories, which validates the superiority and effectiveness of our model.

**Fig.9 Confusion matrix of three models in the TrashNet dataset. (a)ResNet-50 (b)ResNet-50-B (c)Ours.**

Secondly, we use the Grad-CAM technique to visually highlight the regions that the proposed model focuses on during the classification process. The basic principle of Grad-CAM is to calculate the contribution of each feature map to the final classification result using the gradient information from the neural network with the following steps:

1. Compute the gradient: For the input image X, we calculate the gradient of the last convolutional layer through backpropagation.
2. Weight the feature maps: We multiply each feature map's gradient by its corresponding activation value, obtaining each feature map's contribution to the final classification.
3. Generate the Grad-CAM heatmap: We multiply the weighted feature maps by their activation values and sum them up to generate a heatmap. Then, we apply a ReLU activation to the heatmap to retain only the positive contribution and remove negative areas.
4. Overlay the heatmap on the original image: The generated heatmap is resized to match the size of the original image and overlaid on top of it. This allows us to visually see which areas of the image have the most influence on the model’s decision.

In the heatmap, red or yellow areas represent the key regions the model focuses on, which significantly impact the model's classification prediction. Since the principles of Grad-CAM are widely accepted and easy to understand, we have not discussed its theoretical details in the paper.

We have added an experimental section. Specifically, we randomly selected 12 images from the TrashNet test set, generated the corresponding heatmaps using Grad-CAM, and overlaid them on the original images to visually demonstrate the key areas the model focuses on during the classification process, as shown in Fig.5. It can be seen that the proposed model effectively focuses on the objects in the image, demonstrating its ability to capture critical information, thereby achieving high classification accuracy.

**Fig.5 The heatmap of the model's focused regions based on Grad-CAM.**

Finally, in response to your suggestion to compare with the latest methods, we reviewed the relevant literature and found that there has been relatively little research on deep learning-based garbage image classification using the TrashNet dataset in recent years. Therefore, we included results from two relevant papers on the TrashNet dataset and the results from the paper we cited earlier [49] in Table 4 for comparison. We have incorporated the latest research findings to ensure a fair evaluation of our model within the current research context. We believe the comparison in Table 4 covers the major studies related to the TrashNet dataset that are currently available.

We hope these additional experiments and analyses further enhance the depth and clarity of the paper. We appreciate your valuable suggestions and look forward to your further feedback.

**Reviewer#3, Concern#7:** The discussion effectively relates findings to research questions but could better acknowledge limitations, such as dataset size or potential overfitting risks due to TrashNet's limited diversity.

**Author response:** Thank you for your valuable comments on the "discussion" section. We have added a new paragraph in the "discussion" chapter to provide a more detailed explanation of our study's limitations. We believe these additions enhance the comprehensiveness of the paper, more accurately reflect the study’s limitations, and offer useful directions for future research. Thank you again for your insightful feedback, and we look forward to your further suggestions.

**Reviewer#3, Concern#8:** The conclusion succinctly summarizes key findings but does not provide actionable recommendations or insights into future research directions.

**Author response:** Thank you for your positive feedback on our "conclusion" section. In response to your suggestion, we have revised the "conclusion" and added a paragraph discussing future research directions. We believe these additions will provide readers with more actionable and forward-looking insights, further improving the completeness of the paper. Thank you for your valuable input, and we look forward to your further feedback.

**Reviewer#3, Concern#9:** References are relevant and formatted correctly but should include more recent studies to reflect the latest advancements in garbage classification.

**Author response:** We sincerely appreciate your recognition of our references' relevance and correct formatting. In response to your suggestion that the references should include more recent studies, we have made corresponding revisions and additions to the literature review section. Reviewing the literature, we noticed relatively limited research on deep learning-based garbage image classification using the TrashNet dataset. Therefore, we have added the following two representative papers to reflect the latest advancements in this area:

*[50] Shi C, Tan C, Wang T, et al. (2021). A multi-layer hybrid convolutional neural network for waste classification. Applied Sciences, 11(18): 8572.*

*[51] Hossen M M, Majid M E, Kashem S B A, et al. (2024). A reliable and robust deep learning model for effective recyclable waste classification. IEEE Access.*

At the same time, we have also observed progress in the research on deep learning-based garbage image classification models using custom datasets. Therefore, we have supplemented the following papers:

*[52] Alsubaei F S, Al-Wesabi F N, Hilal A M (2022). A deep learning-based small object detection and classification model for waste management in smart cities and IoT environments. Applied Sciences, 12(5): 2281.*

*[53] Liu W, Ouyang H, Liu Q, et al. (2022). Waste image recognition based on transfer learning and model fusion. Engineering Mathematics Problems, 2022(1): 4793555.*

*[54] Li Y, Liu W (2023). Deep learning-based waste image recognition algorithm. Applied Nanoscience, 13(2): 1415-1424.*

We believe these additional references better reflect the latest research trends in the field and provide a stronger theoretical foundation for our study. Thank you again for your valuable suggestions, and we look forward to your further feedback.

**Reviewer#3, Concern#10:** Writing quality is generally good, with minor grammatical errors and occasional verbosity that could be streamlined for better readability.

**Author response:** We appreciate your positive feedback on our manuscript's writing quality and valuable suggestions. We have carefully reviewed the grammatical errors in the text and made corresponding revisions to improve the fluency of the language. In addition, we have streamlined some verbose sections to enhance readability and conciseness. We hope these revisions meet your expectations and improve the paper's overall quality.

Again, we would like to express our sincere gratitude for your time and effort, as well as your support and valuable feedback on our research. We look forward to your final review and hope to receive your approval.
